# Preparation, properties and applications of magnetic nanoparticles

**DOI:** 10.3762/bjnano.1.4

**Published:** 2010-11-22

**Authors:** Ulf Wiedwald, Paul Ziemann

**Affiliations:** 1 Institut für Festkörperphysik, Universität Ulm, 89069 Ulm, Germany

Though studies on small particles of various materials go way back before Nanoscience was emerging, this new interdisciplinary branch of science not only re-termed them into nanoparticles (NPs), but also lead to a dramatically enhanced interest in this type of nanoscaled material with an often given though arbitrary upper diameter limit of 100 nm [1]. As a natural consequence of this worldwide growing interest, the toolbox for preparing NPs has been amazingly broadened including now both, physics and chemistry related approaches. Furthermore, the meanwhile widely accepted distinction between top down and bottom up preparational methods can be applied to the fabrication of NPs as well. Examples for top down approaches are sculpting NPs out of a previously deposited thin film by e.g. Focused Ion Beam techniques [2] or evaporating/sputtering/laser ablating the desired material through nanomasks as e.g. provided by close packed or etched colloidal particles [3–4]. These methods can be applied even if one aims at spherical NPs by subsequent heating resulting in a surface minimizing dewetting process on top of an appropriately chosen substrate [5].

Bottom up approaches mainly can be divided into gas phase condensation [2] or various wet chemical routes [1]. Besides scientific curiosity, the motivation behind all these preparational efforts is certainly to obtain NPs optimized for specific applications. However, as often when surprising new properties are observed for materials, theoretical description and understanding demand for a high reproducibility of the experimental results and, thus, for an optimized sample quality allowing to address specific theoretical questions by corresponding experiments. Such a development can clearly be observed for all types of NPs as well. Indeed, the mere preparation of NPs with broad distributions of size, shape and composition is no longer sufficient. This trend will be briefly demonstrated for a well defined subclass of NPs, the *magnetic* NPs, which are in the focus of the present Thematic Series entitled *“Preparation, properties and applications of magnetic nanoparticles”*.

While the most notable feature of magnetic NPs, their superparamagnetic behavior, has already been reported by Neel as early as 1949 [6], this phenomenon remained rather unnoticed by the broader physics community. Due to the rapid development of information technology, however, with its ever growing demand for higher storage densities, miniaturization of magnetic bits became an important issue. Thus, roughly since the mid nineties, when the corresponding road map of storage density opened the horizon towards the magic Tbits/inch^2^ goal, the idea of using densely packed magnetic NPs for that purpose immediately brought back and spread the awareness of their related superparamagnetic behavior. Trivially, data storage at ambient temperature over a time scale of typically a decade is not at all compatible with superparamagnetism.

A natural way out of this problem is to look for materials exhibiting an as high as possible magnetocrystalline anisotropy which suppresses fluctuations of the effective magnetic moment of the NPs [7]. For binary alloys like FePt or CoPt, which are well known for their high anisotropies, this approach should allow enhancing the related blocking temperatures significantly above ambient even for corresponding NPs with diameters of 3 nm if the particle anisotropy keeps its bulk value. In practice, however, that is exactly the problem: For NPs significantly smaller anisotropies or, closely related to that, smaller coercive fields are generally found shifting their tolerable size for data storage up to approximately 7 nm. Though coupling of NPs to an antiferromagnetic support layer via exchange bias may offer an alternative remedy of the problem [8], a thorough understanding of the reduced magnetocrystalline anisotropy in nanoparticles is still missing. The route to such an understanding is, however, tedious and requires highest possible particle quality. It turns out that the application of magnetic NPs for data storage indeed implies the most stringent conditions like narrow distributions of particle size and, in case of alloys, of chemical composition as well as stability. Furthermore, in order to write and read the information into and from a NP, its position has to be accurately defined. This demands highly ordered arrays of the NPs, in the ideal case two-dimensionally periodic arrangements. To this end, highly reliable and reproducible self-organizing processes are sought allowing a high throughput at a tolerable price.

Preparation, however, has to be accompanied by a strict quality control including the particles’ arrangement, their shape and structure as well as their magnetic properties. While corresponding characterization tools are available to extract averaged information on a particle array supported on top of a suitable substrate, direct measurements on single particles are either often hampered by a relative small statistical significance like in case of High Resolution Transmission Electron Microscopy (HR-TEM) and Spin Polarized Scanning Tunneling Microscopy (SP-STM) or the necessary lateral resolution is only on the verge of being approached as in case of synchrotron-based microscopy methods like Photoemission Electron Microscopy with element-specific magnetic contrast (X-PEEM) [9]. Thus, at the moment one mostly has to rely on ensemble averages and, consequently, this again poses strong requirements on preparation with respect to narrow distributions whenever size dependent properties play a role. This close interrelation between preparation, properties and control is certainly emphasized in the present thematic series reflecting also the fact that the field of magnetic NPs is maturing.

Finally, this series addresses applications of magnetic NPs. Besides the obvious and most demanding one related to magnetic data storage, the use of such particles in the much broader field of sensor technology is described including especially medical diagnostics.

We hope that the selection of review articles as well as full research papers we have chosen is found consistent and useful by the readers interested in that topic and we would like to thank all colleagues for their valuable contributions.

Ulf Wiedwald and Paul Ziemann

Ulm, November 2010
